# Minor surgical procedures and musculoskeletal injections by primary care physicians - an Israeli experience

**DOI:** 10.1186/2045-4015-3-12

**Published:** 2014-03-25

**Authors:** Sasson Menahem, Andrey Nazarenko, Pesach Shvartzman

**Affiliations:** 1 Department of Family Medicine, Faculty of Health Sciences, Siaal Research Center for Family Medicine and Primary Care, Ben-Gurion University of the Negev, POB 653, Beer-Sheva 84105, Israel; 2 Clalit Health Services – South District, Beer-Sheva, Israel

**Keywords:** Health maintenance organization, Minor surgical procedures, Musculoskeletal injections, Primary care physician, Barriers

## Abstract

**Abstarct:**

**Background**

Since minor surgical and musculoskeletal problems are commonly seen in primary care, primary care physicians are expected to possess the skills required to perform minor surgical procedures (MSP) and musculoskeletal injections (MSI).

**Objective**

To evaluate the performance of MSP and MSI by primary care physicians in the Southern District (Negev) of Clalit Health Services (CHS) health maintenance organization (HMO) in Israel.

**Methods**

A structured self-report questionnaire was sent to all 277 primary care physicians, other than pediatricians, working in the Southern District (Negev) of CHS HMO.

**Results**

One hundred fifty one of the 277 questionnaires (54%) were completed and returned. Sixty five percent of the primary care physicians perform any MSP and 46% perform any MSI. The main barriers reported for performing MSP and MSI were lack of time (74% and 66%, respectively) and training (41% and 60%, respectively). Forty percent of the physicians cited remuneration as a potential motivating factor. A logistic regression model showed that male physicians and physicians who work full or part-time in rural areas, are more likely to perform MSP (Odds ratio 2.12 and 2.24, respectively). Male physicians, especially board-certified family physicians, are more likely to perform MSI (Odds ratio 2.86 and 7.0 respectively).

**Conclusion**

MSP and MSI are practiced by only some primary care physicians. HMOs and individuals responsible for designing training curricula in family medicine and primary care can encourage primary care physicians to perform MSP and MSI by providing courses, specific compensation, and dedicated time. This can strengthen the bond between primary care physicians and patients, reduce waiting time for patients, and save money for HMO’s.

## Background

Skin and subcutaneous lesions (nevi, fibromas, lipomas), lacerations, ingrown nails and abscesses [1-3], and musculoskeletal problems like arthritis, bursitis, trigger points, entrapment neuropathies and tendinitis, [4-13] are frequent complaints encountered by primary care physicians, who are expected to have the basic treatment skills required to perform minor surgical procedures (MSP) and musculoskeletal injections (MSI). There are several advantages to performing MSP and MSI in the primary care setting [14-16] including: (a) anxiety reduction, since the procedure is performed by the patient’s family physician and not a stranger, (b) greater convenience for the patient, due to proximity to and familiarity with the clinic, (c) financial savings, since procedures conducted in primary care clinics are less expensive than hospital procedures, and (d) shorter waiting times. Conducting these procedures in the primary care setting can improve the physician-patient relationship and enable primary care physicians to increase the spectrum of their work, enhance their work satisfaction, and help them avoid burnout.

The performance of MSP requires appropriate equipment and is time-consuming. In addition, MSP and MSI require prior training. Without specific compensation for these procedures, primary care physicians may not be motivated to perform them. A survey of 309 primary care physicians from Northern Ireland, published in 2003, reported that only 46% of the physicians performed any MSI [17], and a survey from Croatia reported that fifty percent of the physicians did not perform any surgical procedures on their patients, irrespective of their prior training [18].

Primary care services are provided in the Israeli health system by four health maintenance organizations (HMOs). Currently, there are no formal MSP and MSI courses during the residency program and no postgraduate instructional courses on the subject in Israel.

The goal of the present survey was to evaluate the extent to which primary care physicians treating adults in southern Israel, who work for the largest HMO in Israel - Clalit Health Services (CHS HMO), perform MSP and MSI.

## Methods

### Setting

At the time of this study, 54% of the Israeli population (3,774,600 enrollees) was insured by CHS HMO. The Southern District (Negev) of CHS HMO insures about 280,000 persons above the age of 16 in southern Israel.

Approximately 2,000 primary care physicians work in one of the four HMOs in Israel. About half of them are board certified in family medicine. The rest are general practitioners or board certified internists. Most of the primary care physicians are salaried employees. In CHS HMO primary care physicians are not compensated for the MSP and MSI that they perform. Each primary care physician has a practice of about 1300 persons that he/she is responsible for.

### Selection of study subjects

During the year 2010 a self-report questionnaire was sent by HMO internal mail to all 277 primary care physicians who treat adults in the Southern District (Negev) of CHS HMO. The human resources section of the HMO’s district administration provided the list of physicians. Two months later a reminder was sent to physicians who had not responded. The study included residents and specialists in family medicine, general practitioners without formal training in family medicine, and specialists in internal medicine.

### Measurements

The questionnaire included socio-demographic data, questions relating to the performance of MSP and MSI, and barriers to their performance.

### Statistical analysis

Data was analyzed using the SPSS statistical software package (SPSS, Inc., Version 17.0, Chicago, IL). T-tests and chi-square tests were used to assess statistically significant differences for continuous and categorical variables, respectively. Two-tailed p-values below 0.05 were considered statistically significant. Two logistic regression models were developed to predict MSP and MSI. The models included demographic variables that were significantly associated with MSP and MSI in bivariate data analysis.

The study was approved by the IRB of the Meir Medical Center, Kefar Saba, Israel.

## Results

### Study population compared to all primary care physicians in the southern district (negev) of the CHS HMO

Of the 277 physicians who received the questionnaires by email, 151 returned them (54.5% response rate). The characteristics of the study physicians are presented in Table 1. The study population included significantly more graduates of israeli medical schools, more specialists in family medicine, more veteran physicians, and more physicians who worked in larger sized practices compared to the overall physician population in the district. Fifty nine percent of the physicians in the study population indicated that their main work place was in an urban clinic.

**Table 1:** Demographic characteristics of the study population (N = 151), compared with all primary care physicians in the Negev district

	**Study population (n = 151)**		Primary care physicians in the CHS* Negev district** (n = 277***)		**P value**
	**N**	**%**	**N**	**%**	
**Gender**					
Male	73	48.3%	129	46.6%	0.725
Female	78	51.7%	148	53.4%	
	151		277		
**Age**					
Mean ± SD	48.37 ± 8.95		48.5 ± 8.79		0.557
Range	32-65		31-68		
	149	(mis = 2)	277		
**Country of birth**					
Israel	35	24.1%	67	24.2%	0.99
Other	110	75.9%	210	75.8%	
	145	(mis = 6)	277		
**Country of medical studies**					
Israel	34	23.6%	39	14.1%	0.014
Other	110	76.4%	238	85.9%	
	144	(mis = 7)	277		
**Professional status**					
Specialist in family medicine	86	57.0%	104	37.6%	<0.0001
Resident in family medicine	25	16.6%	54	19.5%	
General practitioner	22	14.6%	105	37.9%	
Expert in internal medicine	14	9.3%	14	5.0%	
Other	4	2.6%	0	0.0%	
	151		277		
**Years in practice as a physician**					
1-10	15	10.4%			<0.0001
11+	129	89.6%			
Mean ± SD	23.49 ± 9.54		10.35 ± 12.28		
Range	3-43		0-38		
	144	(mis = 7)	277		
**Average number of patients per physician**					
Mean ± SD	1476 ± 500		1340 ± 445		0.001
**Range**	250-3000		250-3723		
	131	(mis = 20)	277		

*Southern District (Negev) of Clalit Health Services-health maintenance organization.

**Pediatricians were not included.

*** The list of physicians was extracted from the human resources section of the HMO’s administration.

### Primary care physicians’ performance of MSP and MSI

Sixty five percent of the primary care physicians reported that they perform any MSP, and 46% perform any MSI. Table 2 shows the spectrum of MSP and MSI performed by the study physicians. The performance rate of MSP and MSI was lower than the actual training rate of the physicians.

**Table 2:** A comparison of physicians trained to perform minor surgical procedures and musculoskeletal injections and those who actually do so

	**Trained (n = 151)**			**Actually performed**		
	**n**	**%**	**mis**	**n**	**%**	**mis**
**Minor surgery procedures**						
Suturing of wounds	88	58.7%	1	72	48.0%	1
Drainage of abscesses	78	52.3%	2	76	51.0%	2
Removal of ingrown nails	78	52.0%	1	42	28.4%	3
Excision of cyst or subcutaneous nodule	44	29.3%	1	31	20.8%	2
Excision of nevi	25	16.7%	1	15	10.0%	1
Excision of skin tumors	17	11.3%	1	9	6.1%	3
Drainage of thrombosed hemorrhoids	14	9.3%	1	9	6.1%	3
**Musculoskeletal injections**						
Tennis elbow	85	57.4%	3	52	34.9%	2
Drainage of knee effusions	75	51.0%	4	42	28.4%	3
Plantar fasciitis	75	51.0%	4	54	35.8%	0
Rotator cuff tendinitis	72	48.6%	3	30	19.9%	0
Trigger finger	66	44.6%	3	44	29.1%	0
De Quervain’s tenosynovitis	65	43.9%	3	34	22.7%	1
Golfer’s elbow	59	40.1%	4	34	22.5%	0
Prepatellar bursitis	59	40.1%	4	31	21.4%	6
Acromioclavicular osteoarthritis	57	38.5%	3	23	15.2%	0
Olecranon bursitis	57	38.5%	3	38	25.3%	1
Carpal tunnel syndrome	54	37.0%	5	31	20.5%	0
Biciptal tendinitis	53	35.8%	3	24	15.9%	0
Trochanteric bursitis	35	23.6%	3	23	15.2%	0
Meralgia parasthetica	31	20.9%	3	17	11.3%	1
Dupuytren’s contracture	29	19.6%	3	17	11.3%	1
Ischiogluteal bursitis	22	15.0%	4	11	7.4%	2
Other - cutaneous nerve entrapment	not asked			2	1.3%	0
Other - osteoarthritis of knee	not asked			2	1.3%	0
Other - trigger points	not asked			3	2.0%	0
Other - abdominal or thoracic wall	not asked			1	0.7%	0

Primary care physicians who do not perform MSP usually refer most cases to specialists, except for lacerations that require suturing, which are referred, in 67% of the cases, to the emergency room. The other cases are referred to another primary care physician who does perform suturing. For abscess drainage 61% are referred to a specialist surgeon and 34% to the emergency room. For the different MSI, 80-90% of the patients are referred to a specialist if the primary care physician does not do it. Physicians who performed MSP were more likely to perform MSI (p < 0.0001).

### Facilitating factors and barriers to MSP and MSI performance

The reasons reported for performance or non-performance of minor surgical procedures and injections by the participating physicians are shown in Table 3. Factors reported by all the participating physicians that could facilitate their performance of MSP and MSI included: (a) having dedicated time for these procedures (70%), (b) having undergone a training course (58%), (c) having participated in a demonstration on patients (52%), (d) receiving remuneration (42%), and (e) training on mannequins (40%).

**Table 3:** Reasons for performance or nonperformance of minor surgical procedures and musculoskeletal injections

	Minor surgical procedures (N* = 98)			Musculoskeletal injections (N* = 70)		
	**n**	**%**	**MIS**	**n**	**%**	**MIS**
**Main reason for performance (more than one answer possible0029)**						
It’s an integral part of a family physician’s work	65	75.6%	12	55	84.6%	5
Decreased waiting time for the procedure, compared to secondary care	58	67.4%	12	3	4.6%	5
An opportunity to vary the family physician’s work, thus increasing job satisfaction	54	62.8%	12	50	76.9%	5
Increased patient confidence in the clinic’s medical staff	47	54.7%	12	42	64.6%	5
Decreased patient anxiety level because of treatment by a familiar and trusted staff	36	41.9%	12	32	49.2%	5
Procedures performed in the primary care clinic are less expensive than in secondary care	22	25.6%	12	13	20.0%	5
Other - Immediate help to the patient	Not asked			5	7.1%	0
**Main reasons for nonperformance (more than one answer possible)**						
Lack of time	104	74.3%	11	85	65.9%	22
Lack of knowledge	57	40.7%	11	78	60.0%	21
Lack of equipment	48	34.5%	12	27	20.8%	21
Fear of complications	48	34.3%	11	52	40.0%	21
Other specialists are more qualified to perform the procedures and injections	46	32.9%	11	57	43.8%	21
Lack of remuneration	34	24.5%	12	25	19.5%	23
No personal interest	15	10.7%	11	15	11.5%	21
It’s an integral part of the family physician’s work	10	7.1%	11	12	9.2%	21
Negative experience in the past	5	3.6%	11	5	3.8%	21
Lack of experience	2	1.3%		1	0.7%	

*Only by the performing physicians.

Male physicians were more likely than female physicians to perform MSP (75% vs. 55%, p < 0.007) and MSI (55% vs. 38%, p < 0.032). Physicians born in Israel performed more MSP than physicians born elsewhere (89% vs. 58%, p < 0.001). While this was also the case for MSI, the difference did not reach statistical significance. Graduates of israeli medical schools performed more MSP and MSI than those who graduated from medical schools outside of israel. Specialists in family medicine performed more MSI than residents in family medicine, general practitioners, and specialists in internal medicine (63% vs. 33%, p < 0.0001). This was also the case for MSP, but the difference did not reach statistical significance. Physicians practicing in rural areas performed more MSP than physicians practicing only in urban areas (78% vs. 58%, p < 0.01), while the difference was not significant for MSI. Age, seniority as a physician, the number of patients in the practice, and the number of patients seen in the course of a day were not associated with the performance of MSP and MSI.

Table 4 presents a logistic regression model predicting performance of MSP and MSI. All demographic variables that were significantly associated with MSP and MSI in the bivariate analysis were entered into this model. Age, which was not significantly associated with MSP and MSI, was added to the model nonetheless for variable stratification. Being a male physician and working in a rural clinic (full or part-time) predicted performance of MSP (OR = 2.12, 95% CI 1.04-4.35 and OR = 2.24, 95% CI 1.01-4.99, respectively). Being a male family physician, and especially being board-certified in family medicine predicted performance of MSI (OR = 2.87, 95% CI 1.35-6.10 and OR = 7.01, 95% CI 3.15-15.58, respectively).

**Table 4:** Logistic regression model predicting the performance of MSP and MSI by primary care physicians

**Variable**	**OR**	**95% CI**	**P-value**
**Minor surgical procedures model**			
Gender (male)	2.124	1.036-4.351	0.04
Age	1.004	0.961-0.044	0.859
Main practice in rural setting	2.245	1.011-4.987	0.047
**Musculoskeletal injections model**			
Gender (male)	2.868	1.348-6.101	0.006
Age	0.961	0.921-1.003	0.07
Specialty in family medicine	7.009	3.153-15.577	<0.0001

## Discussion

Although all primary care physicians frequently encounter medical problems requiring the performance of MSP and MSI, and more than two thirds of the participating physicians stated that MSP and MSI should be an integral part of the family physician’s work, about one third do not perform MSP and one half do not perform MSI. The most common MSP that primary care physicians were trained to perform, and which about half actually do perform were suturing of lacerations and draining of abscesses. The main reasons that those who performed MSP did so were to decrease waiting times for procedures for patients and to add variety to the family physician’s work. The main barriers to performing MSP and MSI, as cited by 50-70% of the physicians, were a lack of dedicated time and the absence of hands-on training programs. Male physicians and physicians working in rural clinics were twice as likely to perform MSP as female physicians and physicians working in city clinics. Male physicians were more likely to perform MSI than female physicians by a factor of 2.86. Board certified physicians in family medicine were seven-fold more likely to perform these procedures than the other physicians in the study.

### Comparison with existing literature

Several surveys have addressed the issue of MSP in primary care. Two Israeli family physicians reported their experience with surgical procedures in their clinics over a 22-month period in 1984–5. They treated both rural and urban patients. In the rural setting they performed more elective surgical procedures than in the urban setting. More urgent surgical procedures were performed in the urban clinics [15]. Their complication rate was about 3%, which is the expected rate [16]. The most common surgical procedures included suturing lacerations, excision of skin lesions and sebaceous cysts, removal of nails, draining of abscesses, removal of foreign bodies, and drainage of thrombosed hemorrhoids.

A prospective survey from the United Kingdom checked the cost effectiveness of minor surgery with remuneration in general practice compared to hospital practice. Minor surgery in general practice was cost effective but general practitioners sent a smaller proportion of specimens to a histopathology laboratory, incorrectly diagnosed a larger proportion of malignant conditions as benign and inadequately excised 5% of the lesions [19].

Another prospective randomized comparison of minor surgery for 568 patients between primary and secondary care was conducted in the United Kingdom. Again, minor hospital surgery was of slightly better quality with a difference that reached statistical significance. However, the clinical importance of the difference was uncertain and the cost was higher. The complication rate was similar in both groups except for wound infection, which was higher in primary care minor surgery. Patients were more satisfied doing procedures in primary care setting because it was more convenient [14].

The results of a survey from Spain showed that the average waiting time for procedures performed by family physicians was lower by a mean of 45 days than surgeons, without any significant difference in effectiveness [20]. Several surveys from Canada reported similar findings to those of the present study. They also reported that primary care physicians in rural areas perform more surgical procedures than those in urban areas [21,22]. In a survey from Ontario Canada, 79 family physicians were interviewed. The overall self-reported performance rates were 63.3% for dermatological excisions, 43% for knee injections, and 31.6% for shoulder injections. These rates were higher than in our survey (20.8%, 28.4%, and 19.9% respectively). Similar to our findings, the main barriers to performing these procedures were lack of updated skills and lack of time (about 50%) [23]. In Saudi Arabia, a randomly selected group of 231 primary care physicians working in Riyadh health centers completed a confidential questionnaire about their performance of minor surgical procedures. The results were similar to ours with 74% of the physicians performing some sort of minor surgery. Physicians living in remote areas performed more minor surgery compared to other areas and male physicians performed more minor surgery than female physicians (p = 0.05) [24].

In a study from Croatia, the effect of a surgical training course on the performance of minor surgical procedures was evaluated one year later in a group of 59 family physicians. There was a statistically significant increase in the number of minor surgical procedures performed, which almost doubled from baseline. Male physicians performed significantly more surgical procedures than female physicians, before and after the course. As in our study, there was no association between the number of procedures performed and the age of the participating physicians or their length of employment, but in contrast to our study there was no difference between urban and rural clinical settings. Fifty percent of the physicians did not perform surgical procedures irrespective of whether they participated in the course [18], compared to one third in the present study.

Similarly, several surveys have addressed MSI performance by primary care physicians. A survey from the United Kingdom explored joint and soft tissue injections by 251 general practitioners. Factors associated with higher levels of injection activity were: male gender, more than 10 year’s experience, a special interest in rheumatology or orthopedics, and working in a rural or mixed practice. The most important barriers to carrying out injections were lack of practical training, lack of confidence, and inability to maintain skills. The most injected musculoskeletal problems were tennis elbow, knee joint and glenohumeral joint [25].

In a survey among 798 primary care physicians from Ontario, Canada who completed a questionnaire about their level of confidence in treatment of musculoskeletal disorders rural physicians were more confident than urban physicians about doing a joint injection/aspiration (Odds ratio 2.24) [26]. In a study conducted in Northern Ireland 46% of the 309 participating physicians did not perform MSI at the time of the study. Five percent of the primary care physicians carried out most of the injections done in the community. Injections into the shoulder, knee and lateral epicondyle were the most commonly performed. The physicians preferred to train on “real patients” rather than on mannequins. The barriers to performing injections included (in descending order): inability to maintain injection skills, inability to make the correct diagnosis, medico-legal concerns, concerns about complications, cost/time involved in training, time needed to do the procedure, lack of evidence about efficacy, and lack of personal interest [17]. In our survey lack of time was the main barrier.

In a randomized study from Northern Ireland of two different training programs for general practitioners in the techniques of shoulder injection, physicians that received training on real patients were significantly more confident in performing injections than physicians who were trained using mannequins [27]. Two more studies from the United Kingdom and the Netherlands showed that training programs for primary care physicians on shoulder injections techniques increase their performance rate [28,29]. Another survey of primary care internists from the United States showed that a training program in outpatient primary care could increase MSI performance [30].

A survey of 298 physicians working in a primary care setting in Riyadh, Saudi Arabia reported their MSI experience. The conclusion was that many physicians working in primary care settings in Saudi Arabia refer patients who require musculoskeletal injections to specialists for consultation, even though treating these patients at the primary care level is more time- and cost effective [31].

### Interpretation of the study results in relation to existing literature

In our survey, as well as those reported from other countries, MSP and MSI performance rates were higher in male physicians and those who work in rural areas. The best performance improvement reported in the literature resulted from a course in which physicians practiced on actual patients. The physicians in the present study ranked setting aside dedicated procedure time and a training course as the most important facilitating factors, while they ranked live demonstrations on patients as third.

The performance rate for MSP and MSI was not associated with the physician’s age, years in practice, the number of patients in the practice, or the number of patients seen by the physician over the course of a day. This consistent finding suggests that those primary care physicians who are used to performing MSP and/or MSI continue to do so as they gain experience and confidence and manage to find the time despite their heavy work burden.

### Strengths and limitations

Our study has some limitations. First, only 54% of the eligible physicians in the Southern District (Negev) of CHS HMO responded to the survey. Second, self-report is less accurate than actual measurement of performance and physicians may overestimate their performance. Third, those who answered the questionnaire could be more interested in the subject and tend to perform procedures more than those who did not. Forth, the study was limited to the south of Israel and included physicians from only one HMO, albeit the largest in the region. Potential socio-demographic differences between all primary care physicians in the Southern District (Negev) CHS HMO and those who participated in the study could affect the results, since our study population consisted of a higher percentage of specialists in family medicine and graduates of Israeli medical schools compared to all primary care physicians in the south. Thus, the performance rate for all primary care physicians might actually be lower than the observed rate in this study. Sending specimens to histopathological examination could be a barrier to MSP performance that was not addressed in the questionnaire.

However, the results of the present survey are similar to those of studies from other countries, a finding that strengthens the reliability of the results for our region.

### Health policy implications of the findings

More than two thirds of the primary care physicians in the study stated that MSP and MSI should be an integral part of their job. In actual practice, the overall performance rate was low. By providing courses for residents and post-graduates and providing appropriate compensation, time and equipment, the performance of MSP and MSI could be increased. This would save money for the HMOs, which function in a setting of an ongoing financial crisis in the healthcare system in Israel and in other countries where specialist fees are much higher than those of primary care physicians, even though they have longer waiting times.

## Conclusion

Although the majority of primary care physicians state that MSP and MSI should be an integral part of their work and its performance is cost effective, it is practiced by primary care physicians at lower rates than expected.

### Implications for clinical practice

HMOs and individuals responsible for training curricula in family medicine could encourage primary care physicians to perform MSP and MSI by conducting dedicated courses, with practical “hands-on” experience on actual patients, specific remuneration, and dedicated time. An emphasis should be placed on women and primary care physicians working in urban areas. These procedures should be taught in the framework of residency programs as well as postgraduate continued medical education (CME) in primary care.

Implementation of these recommendations could save money for the healthcare system and time for patients, while strengthening the bond between primary care physicians and their patients.

## Competing interests

The authors declare that they have no competing interests.

## Authors’ contributions

All authors read and approved the final manuscript.
